# Over-Pressure in Children

**Published:** 1889-06

**Authors:** 


### Over-Pressure in Children.

In a note recently published by Professor Charcot on "over-pressure," the author asserts that intellectual or cerebral over-work does not exist in children under sixteen years of age. A child, he says, can make only the amount of intellectual effort of which he is capable. If he has programmes too overcharged to fulfill, he simply does not fulfill them; if one insists on cramming his memory with crude facts, no result whatever is obtained; but this does not in any way affect the brain of the child, the passiveness of which is complete, and the indifference absolute. On the other hand, according to the Professor, "over-pressure manifests itself only in youths above sixteen or eighteen years. It is characterized by a number of nervous troubles, principally by a pain in the occipital region, which extends down to the back of the neck, and goes up again in front of the ears. This over-pressure is seen in pupils of the superior branches of study, in men of letters who write much, in political men who are or who believe themselves to be overwhelmed with responsibilities, in men of business, etc., who lose their sleep, but never in the pupils of our *lycees* and colleges."—*Lancet*, Feb. 23, 1889.

